# Recurrence rates in dogs with GDV treated with gastric decompression versus dogs treated with gastric decompression and gastropexy

**DOI:** 10.18849/ve.v10i1.695

**Published:** 2025-02-10

**Authors:** Costel Zagan

**Keywords:** canine, decompression, dogs, gastric dilatation, gastropexy, gdv, stomach

## Abstract

**PICO question:**

In dogs diagnosed with gastric dilatation and volvulus (GDV), does gastric decompression and gastropexy reduce the risk of recurrence of dilatation, compared to dogs undergoing gastric decompression alone?

**Clinical bottom line:**

**Category of research:**

Treatment.

**Number and type of study designs reviewed:**

Four papers were critically reviewed. Two papers were retrospective studies, and two were prospective studies.

**Strength of evidence:**

Strong.

**Outcomes reported:**

In dogs with GDV, the risk of recurrence of this disease was much lower when gastropexy was performed.

**Conclusion:**

The studies comparing the recurrence rates in dogs with GDV treated either with gastric decompression (with or without gastric repositioning) or with gastric decompression and gastropexy indicate that gastropexy will significantly reduce the risk of recurrence.

## How to apply this evidence in practice

The application of evidence into practice should take into account multiple factors, not limited to: individual clinical expertise, patient’s circumstances and owners’ values, country, location or clinic where you work, the individual case in front of you, the availability of therapies and resources.

Knowledge Summaries are a resource to help reinforce or inform decision-making. They do not override the responsibility or judgement of the practitioner to do what is best for the animal in their care.

### Clinical scenario

A Cane Corso dog is brought into your practice with an acute onset of non-productive retching abdominal distension, restlessness, and weakness. After diagnosing gastric dilatation and volvulus (GDV), and administering shock treatment, would you only perform gastric decompression or perform gastric decompression and continue with gastropexy?

### The evidence

Four studies (Glickman et al., (1998); Eggertsdóttir et al., (1996); Meyer-Lindenberg et al., (1993); Wingfield et al., (1975)) have been found to fit with the PICO question. All of them investigated the recurrence rate of GDV in two groups of dogs, the group treated with gastric decompression and gastropexy versus the group treated with gastric decompression alone. Based on these papers, this Knowledge Summary has strong evidence.

### Summary of the evidence

**Eggertsdóttir et al. (1996):** Comparison of two surgical treatments of gastric dilatation-volvulus in dogs. **Aim: **Compare the effect of two methods of treatment for gastric dilatation and volvulus (GDV) (gastric decompression vs gastric decompression and gastropexy) in dogs.

Population	Dogs from 16 breeds that were referred to the Department of Small Animal Clinical Sciences, Norwegian College of Veterinary Medicine, between March 1991 and November 1992. Selection criteria: only dogs diagnosed with gastric dilatation and volvulus (GDV) that had not experienced another acute attack of GDV in the last 5 months were included in the study. All breeds, ages, and both sexes were allowed. The time between the acute stage of the disease and arrival at the referral clinic had to be less than 10 days.
Sample Size	31 dogs. The dogs were split into 2 groups: initially, group A had 21 dogs. From this group, 2 dogs were euthanised during surgery due to severe gastric necrosis, therefore 19 dogs were available for the study. Initially, group B had 10 dogs. Five dogs died 1–18 days postoperatively due to GDV-related complications (3 from group A, 2 from group B). Thus 24 dogs were available for follow-up evaluation.
Intervention Details	Group A (n = 16) received decompression and anatomical repositioning of the stomach and a circumcostal gastropexy. Group B (control group: (n = 8)) received the same treatment without gastropexy.
Study design	Prospective open, randomised controlled clinical trial.
Outcome studied	Recurrence rate of GDV. GDV-related complication rate. Death rate in each group within the first year post-GDV. Median survival time.
Main findings (relevant to PICO question)	In group A, 3/16 (19%) dogs died due to GDV-related complications versus 2/8 (25%) in group B. No recurrences in group A (0%). The recurrence rate in dogs that died from group B was 50% (3/6). There was a statistically significant association (P = 0.01) between gastropexy status and risk of recurrence as the dogs that did not have a gastropexy were significantly more likely to have GDV recurrence (50%, 3/6 dogs) than dogs that did (0%, 0/10 dogs) by the end of the study. There was also a statistically significant association (P = 0.04, 95% confidence interval 0.14–0.95) between gastropexy status and survival time as the dogs that had gastropexy performed had a longer median survival time (549 days for dogs in group A) compared to dogs that did not have gastropexy performed (107 days for dogs in group B). The overall death rates within the first year were 32% (6/19) in group A and 80% (8/10) in group B.
Limitations	The follow-up period was relatively short: the median follow-up time for group A was 397 days (mean 430 days) and 106 days (mean 228 days) for group. Small sample population. Three dogs in group A and 4 dogs in group B died within 6 months. The early withdrawals represented by dogs who were euthanised during surgery or died postoperatively reduced the median follow-up time considerably. The sample size of group B was limited due to ethical reasons.

Table note...

**Glickman et al. (1998):** A prospective study of survival and recurrence following the acute gastric dilatation-volvulus syndrome in 136 dogs **Aim: **Measure recurrence and mortality rates, and identify prognostic factors in dogs with gastric dilatation and volvulus.

Population	Dogs that previously had been recruited to participate in a case-control study of risk factors for gastric dilatation and volvulus (GDV) from emergency clinics in the United States (27 clinics in total). All dogs were diagnosed with GDV, based on clinical, radiographic, and surgical findings.
Sample Size	136 dogs were initially recruited. 18 were lost to follow-up, so they were excluded from the study. 33 dogs died or were euthanised within the first 7 days after presentation. 85 dogs remained and were included in the study.
Intervention Details	The dogs received shock treatment and gastric decompression, and then were split into 2 groups: Group 1: dogs that had gastropexy performed (n = 74). Group 2: dogs that did not havegastropexy performed (n = 11).
Study design	Prospective cohort study.
Outcome studied	Recurrence rate of GDV; Age, weight, sex, breed, and neutering status predilection. Median survival time in the two groups. Probability of surviving, respectively dying within the first seven days following an acute GDV episode. Groups of dogs at high risk of GDV relapse following an acute GDV incident.
Main findings (relevant to PICO question)	Group 1: the incidence of recurrence was 4.1% (3/74). Group 2: the recurrence rate was 54.6% (6/11 dogs). The dogs that had gastropexy performed for an acute GDV were much less likely to have a GDV recurrence compared to dogs that did not have a gastropexy (P < 0.001, odds ratio 0.05, 95% confidence interval: 0.01–0.25). The probability of a relapsing episode of GDV was not correlated to the age, weight, sex or neutering status of the dogs. The dogs that had gastropexy had a median survival time of 547 days, whereas in the dogs without gastropexy this was 188 days (P = 0.0001). This is likely related to the high recurrence rate of GDV in dogs from group 2 compared with the dogs from group 1. Most of the dogs presented with GDV to the veterinary clinics were adult (mean of 5.7 years old ±2.8 standard deviation) male entire Great Dane dogs. The probability of surviving the first seven days following an acute GDV episode was not influenced by either the age, weight, sex, neuter status of the cases, the time from onset of clinical signs until presentation to the clinic, or the time from presentation to the time of surgery. The dogs at the highest risk of dying within the first 7 days following an acute episode of GDV were male dogs older than 7 years old, recumbent, depressed or comatose, with gastric necrosis. The dogs at the highest risk of having a recurrence of GDV following treatment of acute GDV were male dogs over 8.9 years of age, between 79–103 lbs that did not have a gastropexy surgery performed.
Limitations	33/136 dogs (24.3%) died or were euthanised within the first seven days. 18/103 surviving dogs (17.5%) were lost to follow-up. There are no details on how the dogs were split into the 2 groups. There is no report of the number of dogs that died from each category in a set amount of time.

Table note...

**Meyer-Lindenberg et al. (1993):** Treatment of gastric dilatation-volvulus and a rapid method for prevention of relapse in dogs: 134 cases (1988–1991) **Aim: **Assess the recurrence rate of gastric dilatation and volvulus in dogs treated with gastric decompression alone compared to dogs treated surgically.

Population	Medical records of dogs with gastric dilatation and volvulus (GDV), treated from January 1988 to April 1991 at the School of Veterinary Medicine’s Clinic of Small Animals in Hanover, Germany were reviewed.
Sample Size	134 dogs were admitted with GDV. Of these, 13 died or were euthanised prior to treatment, therefore 121 dogs were available for the study.
Intervention Details	The remaining 121 dogs were split into 2 groups: Group 1 (n = 88) were treated surgically (gastropexy). Gastropexy was performed by fixing the pyloric antrum to the abdominal wall by incorporating the stomach into the main suture of the linea alba for approximately 5 cm. This suture line included the muscularis of the stomach, without entering the stomach lumen. Group 2 (n = 33) were treated medically (decompression via a gastric tube, without coeliotomy).
Study design	Retrospective case-control study.
Outcome studied	Death rate in each group due to GDV or GDV-related complications. Recurrence rate in dogs with GDV that had medical interventions versus dogs that had surgical interventions. Discharge rate in each group.
Main findings (relevant to PICO question)	In group 1, 14.8% (13/88) of dogs died due to GDV or GDV-related complications and 13.6% (12/88) dogs died due to other causes. The overall death rate in this group was (25/88) 28.4%. In group 2, 18.2% (6/33) of dogs died due to GDV or GDV-related complications and 6.1% (2/88) of dogs died due to other causes. The overall death rate was 24.3% (8/33 dogs). Two owners were not available for a follow-up check in group 1, therefore the recurrence rate in this group was 6.6% (4/61 dogs), whereas in group 2 it was 76% (19/25 dogs). In group 1, the 4 dogs that had a GDV relapse had a death rate of 100%. In group 2, the 19 dogs that had a GDV relapse had a death rate of 68.43% (13/19 dogs). Therefore, the death rate in this group was 63.63% (21/33 dogs). Discharge rate was 71.6% (63/88 dogs) in group 1, whereas in group 2 it was 75.75% (25/33 dogs).
Limitations	The author was not specific about the type of surgery one dog in group 1 had but included it in this ‘surgical treatment’ group. In group 2 it is not stated when the GDV relapsed in the 19 dogs.

Table note...

**Wingfield et al. (1975):** Operative techniques and recurrence rates associated with gastric volvulus in the dog **Aim: **Assess the recurrence rate of gastric dilatation and volvulus in dogs treated with different types of surgery.

Population	87 dogs with gastric dilatation and volvulus (GDV) syndrome were included in the retrospective study of recurrence. These animals were seen at either the Animal Medical Center in New York City, the Small Animal Hospital of the University of Georgia, or the Small Animal Hospital of the University of Missouri.
Sample Size	87 dogs, from which 43 died perioperatively with no GDV recurrence. Thus, 44 dogs were available for recurrence assessment.
Intervention Details	The 44 dogs had a total of 101 surgical procedures performed. The dogs were split into groups depending on the type of surgery they had. Surgical treatment for GDV included gastropexy (35/44 dogs, 79.6%), pyloric surgery (25/44 dogs 56.9%), gastrectomy (6/44 dogs, 13.7%), splenectomy (19/44 dogs, 43.2%), gastric repositioning alone (5/44 dogs, 11.4%), pharyngostomy tube placement (6/44 dogs, 13.6%), and a group where the technique employed was not noted (5/44 dogs, 11.4%).
Study design	Retrospective cohort study.
Outcome studied	The success rate correlated to each type of surgical procedure used to correct a GDV was assessed, where success was referred to the percentage of cases that did not have an episode of GDV relapse during the first 5 months postsurgery.
Main findings (relevant to PICO question)	The dogs that had gastropexy performed had a success rate of 77.2% (27/35 surgeries), thus a 22.9% recurrence rate (8/35). The dogs that had pyloric surgery performed had a success rate of 80% (20/25 surgeries), thus a 20% recurrence rate (5/25). The dogs that had gastrectomy performed had a success rate of 100% (6/6 surgeries), thus no recurrence. The dogs that had splenectomy performed had a success rate of 78.9% (15/19 surgeries), thus a recurrence rate of 21.1% (4/19). The dogs that had gastric repositioning alone performed had a success rate of 20% (1/5 surgeries), thus an 80% recurrence rate. The dogs that had a pharyngostomy tube placed had a success rate of 83.3% (5/6 surgeries), thus a recurrence rate of 17%. The dogs that had surgery for GDV which was not noted had a success rate of 80% (4/5 surgeries), thus a recurrence rate of 20% (1/5).
Limitations	Small number of cases. It is not clear in what treatment categories the 43 dogs that died perioperatively were placed. It is not stated what surgery was done in each patient, as some of the dogs might have had one surgery performed, whereas other dogs might have had multiple surgeries at one time. 23/87 dogs (26.4%) died postoperatively, with no specification if they had or had not a recurrence of GDV at the time of death.

Table note...

## Appraisal, application and reflection

After a thorough search of the literature, four papers have been found to be relevant to this PICO question, answering with a strong level of evidence, as their findings are similar, but the case numbers are not very large.

One retrospective cohort study (Wingfield et al., 1975) reviews 87 dogs with gastric dilatation and volvulus (GDV) at the moment of presentation. Of these 87 dogs, 43 died perioperatively with no GDV recurrence. The rest of the 44 dogs had a total of 101 recurrences of GDV. Of the 101 cases, 35 dogs had incisional gastropexy with 8/35 (22.86%) recurrence rate, and five dogs had gastric repositioning alone with 4/5 (80%) recurrence rate. The rest of 61 cases had other types of surgical procedures performed, not relevant to this Knowledge Summary.

Another retrospective case-control study (Meyer-Lindenberg et al., 1993) looked at 134 dogs diagnosed with GDV at the School of Veterinary Medicine’s Clinic of Small Animals in Hanover, Germany. Of the 134 dogs, six were euthanised immediately after confirmation of the diagnosis, in compliance with the owners’ requests, because of an unfavourable prognosis. Another seven dogs died immediately prior to or during initiation of anaesthesia. Of the remaining 121 dogs, 87 had incisional gastropexy performed. Of these 87 dogs, 63 were discharged with no recurrence. The owners of two of these dogs were not available. Of the remaining 61 dogs, four had a relapse (6.56%). From the 121 dogs treated for GDV, 33 had gastric repositioning alone, with a recurrence rate of 75.76% (25/33).

A third paper, a prospective open, randomised controlled clinical trial (Eggertsdóttir et al., 1996) studied 31 dogs, of which six dogs were admitted with GDV after unsuccessful decompression by the referring veterinarians, the remaining 25 dogs were decompressed and received treatment for shock before referring. There were two dropouts in the study and five dogs died 1–18 days postoperatively due to GDV-related complications. Thus 24 dogs were available for follow-up evaluation. Ten of 16 dogs were alive in group A (treated with gastropexy) after a median time of 397 days (mean 430 days) with a recurrence rate of 0% (0/16). Two of 8 dogs were alive in group B (treated with gastric repositioning alone) after 106 days (mean 228 days). Two dogs were euthanised due to aggressive behaviour, with no GDV recurrence. Therefore, 6 dogs remain in this group for results calculation. The recurrence rate group B was 50% (3/6).

The last relevant paper in this Knowledge Summary, a prospective cohort study (Glickman et al., 1998) assessed 136 dogs referred for GDV treatment from multiple practices. Of these 136 cases with GDV, 33 (24.3%) died or were euthanised during the first seven days following presentation to a veterinary clinic. Of the 103 surviving cases, 18 (17.5%) subsequently were lost to follow-up. Therefore, 74 dogs had incisional gastropexy performed, with a 4.1% (3/74 dogs) recurrence rate, and 11 dogs were treated with gastric repositioning alone, with a 54.6% (6/11 dogs) recurrence rate.

Three studies show similar recurrence rates in dogs with GDV (Meyer-Lindenberg et al., 1993; Eggertsdöttir et al., 1996; Glickman et al., 1998). Rates were much higher (50–76%; 6/11, 3/6, and 19/25, respectively) after intensive medical therapy (such as fluid resuscitation) and gastric decompression alone, compared to the dogs with GDV that had intensive medical therapy and gastropexy performed (0–6.6%; 3/74, 0/16, and 4/61, respectively). Wingfield et al. (1975) showed different rates, but the difference between the 2 groups was still considerable (22.9% recurrence rate in 8/35 dogs with GDV that had gastropexy versus 80% recurrence rate in 4/5 dogs that had gastric decompression alone).

The conclusion of this Knowledge Summary is that in dogs with GDV, the risk of recurrence of this pathology was much lower when the respective dogs received medical therapy for stabilisation, gastric repositioning, and gastropexy, compared to dogs treated with stabilisation and gastric repositioning alone.

## Methodology

**Search Strategy:** 

Databases searched and dates covered	CAB Abstracts on the OVID interface 1973–May 2024 PubMed accessed via the NCBI website 1973–May 2024
Search terms	Cab Abstracts: (dog or dogs or canine or canines or bitch or bitches) or exp dogs/ or exp bitches/ (gastric dilatation or gastric dilatation volvulus or GDV or gastric torsion or stomach volvulus) (gastropexy or ((stomach or gastric) and sutur*)) ((stomach or gastric) and (decompression or deflation or replacing or reposition)) 1 and 2 and 3 and 4 Pubmed: dog OR canine OR bitch gastric dilatation OR gastric dilatation volvulus OR GDV OR gastric torsion OR stomach volvulus gastropexy (stomach OR gastric) AND suture (stomach OR gastric) AND (decompression OR deflation OR replacing OR reposition) 1 AND 2 AND (3 OR 4) AND 5
Dates searches performed:	23 May 2024

Table note placeholder

**Exclusion / Inclusion Criteria:** 

Exclusion	Foreign language papers.Irrelevant to the PICO question.
Inclusion	Any relevant primary veterinary research in English which assessed the recurrence of gastric dilatation and volvulus in dogs after being treated medically and with gastric repositioning, compared to dogs who additionally had gastropexy performed.

Table note placeholder

**Search Outcome:** Caption placeholder

Database	Number of results	Excluded – foreign language papers	Excluded – irrelevant to PICO question	Total relevant papers
CAB Abstracts	23	2	18	3
PubMed	18	1	16	1
Total relevant papers when duplicates removed				4

Table note placeholder

### ORCiD

Costel Zagan: https://orcid.org/0000-0003-0208-3682

### Conflict of Interest

The author declares no conflicts of interest.

## Disclaimer

Knowledge Summaries are a peer-reviewed article type which aims to answer a clinical question based on the best available current evidence. It does not override the responsibility of the practitioner. Informed decisions should be made by considering such factors as individual clinical expertise and judgement along with patient’s circumstances and owners’ values. Knowledge Summaries are a resource to help inform and any opinions expressed within the Knowledge Summaries are the author’s own and do not necessarily reflect the view of the RCVS Knowledge. Authors are responsible for the accuracy of the content. While the Editor and Publisher believe that all content herein are in accord with current recommendations and practice at the time of publication, they accept no legal responsibility for any errors or omissions, and make no warranty, express or implied, with respect to material contained within. For further information please refer to our Terms of Use.
